# Professionalisation or Credentialism? Fifteen‐Year Enrolment Trends in the UK Postgraduate Certificates in Medical Education

**DOI:** 10.1111/tct.70527

**Published:** 2026-09-20

**Authors:** Matthew Bowker, Leah Williams, Michael Atkinson, Chris Tiplady

**Affiliations:** ^1^ School of Medicine University of Sunderland Sunderland UK; ^2^ Northumbria Healthcare NHS Foundation Trust Northumberland UK

**Keywords:** clinical education, credentialism, educator training, Freedom of Information, medical education, postgraduate certificate, workforce

## Abstract

**Background:**

Postgraduate qualifications for clinical educators have proliferated internationally, raising questions about whether this reflects professionalisation or a credentialist response to competitive specialty selection. Postgraduate Certificates in Medical Education (PGCMEs) are established in the United Kingdom (UK), yet no published data describe enrolment over time. This research determined PGCME enrolment trends in the UK universities from 2010 to 2024 and interpreted them against this framing.

**Methods:**

Freedom of Information requests were sent to all the UK public universities offering PGCMEs (*n* = 40), requesting annual enrolment figures. We obtained contextual data on the National Health Service (NHS) workforce size, specialty training competition ratios and application scoring from the NHS England and archived recruitment websites.

**Findings:**

A total of 36 universities (90%) responded. Courses more than doubled (13 in 2010–2011 to 29 in 2024–2025); student numbers rose 41% (1180–1667). Enrolments were stable through the 2010s, jumped in 2020–2021 (1082–1894), then settled at 1600–1700. Against the NHS England workforce, the proportion of doctors enrolled held at about 1%. Training competition rose: general practice ratios from 1.70 to 3.67 and internal medicine training (IMT) from 2.55 to 3.69. The PGCME's IMT scoring weight doubled from 4.7% to 10%.

**Conclusion:**

PGCME provision has grown, but enrolment has not outpaced the expanding medical workforce. The 2020 spike likely reflects pandemic‐era online access and scoring reforms. Aggregate data cannot tell us whether holders teach afterwards or move straight into specialty training, how they funded their studies, whether they completed or what proportion are doctors.

## 1 Background

Medical education is increasingly recognised as a distinct discipline, with growing expectations that those who teach and supervise doctors and medical students possess specialist pedagogical expertise [1, 2]. In the United Kingdom, this expectation is reflected in the number of Postgraduate Certificates in Medical or Clinical Education (PGCMEs) offered by universities and the emergence of professional bodies such as the Academy of Medical Educators (AoME) [3]. Yet despite longstanding interest in the “professionalisation” of medical education [4, 5], there are no published data describing how many healthcare professionals pursue these formal qualifications or how enrolment has changed over time.

This expansion is not unique to the United Kingdom. Postgraduate qualifications in health professions education have grown from a small number of programmes to well over a hundred worldwide in two decades [4]. Two interpretations of this growth coexist in the literature. A professionalisation reading, supported by qualitative work on educator identity formation [6], frames postgraduate qualifications as transformative experiences that develop scholarly, self‐efficacious educators who go on to leadership and scholarship in the field. A credentialist reading observes that postgraduate teaching qualifications have been incorporated into the scoring of competitive specialty selection and that rational applicants accumulate them to remain competitive even where there is no intention of sustained educational practice. Analogous patterns have been described in Australian specialty trainees, nearly half of whom acquire postgraduate qualifications beyond those needed for clinical practice [7]. Recent commentary on the UK specialty selection has argued that the portfolio scoring system is itself driving credential acquisition [8]. These readings are not mutually exclusive, but the balance has implications for whether enrolment growth produces a more skilled educational workforce or a more credentialed applicant pool.

Despite this conceptual interest, no published national data describe the scale of the credentialing pipeline in any health system. We use the United Kingdom as a tractable case, contextualising enrolment against medical workforce growth, specialty training competition and scoring weight. The mechanisms we describe are not specific to the United Kingdom or medicine; we return to wider implications in the discussion.

### 1.1 Aim

The aim of this research is to determine the enrolment trends for Postgraduate Certificates in Medical Education in the UK universities over the last 15 years and to interpret these trends against the framing of professionalisation and credentialism.

### 1.2 Research Question

How have the numbers of students enrolling in postgraduate certificates in medical education in universities in the United Kingdom changed over the last 15 years?

## 2 Methods

### 2.1 Identifying Appropriate Courses

A list of UK universities offering a Postgraduate Certificate in Medical or Clinical Education (PGCME) was compiled in July 2025. This was achieved by searching the course aggregators Prospects.ac.uk and Findamasters.com, as well as reviewing accredited course lists from the AoME and the Clinical Education Research organisation. A final Google search for “postgraduate certificate in medical education” and “postgraduate certificate in clinical education” was performed to a depth of 15 pages to ensure a comprehensive list.

The inclusion criteria specified that the institution must be a UK university offering a postgraduate certificate in medical education or clinical education. Courses with variations in the title, such as ‘medical and health education’, were included, but those without the term ‘medical’ or ‘clinical’ were excluded. This decision was taken as this research focuses on the education of doctors, who would typically attend ‘medical education’ or ‘clinical education’ courses, while other healthcare professionals such as nurses may attend courses such as ‘healthcare professions education.’ While our approach is not totally sensitive or specific, it is sufficient to answer the question of trends over time.

Courses leading to a Postgraduate Diploma (PGDip) or a Master's degree were not included. If a university offered multiple specialised PGCME courses (e.g., ‘PGCME with simulation’), their numbers were merged.

### 2.2 Data Collection

Freedom of Information (FOI) Act requests are an increasingly used method for data collection in medical education research [9]. If the request is appropriate, bodies are required to provide the information within 20 working days.

FOI requests were sent using the platform WhatDoTheyKnow (www.whatdotheyknow.com) from 19th October to 19th November 2025. Requests asked for the number of ‘home’ and ‘international’ students enrolling on the relevant course per academic year since 2010. ‘Home’ student generally refers to individuals who are resident and ‘settled’ in the United Kingdom [10]. ‘International’ students are generally those who do not fall into this category and would usually attract significantly higher tuition fees.

Universities not responding within 20 days received reminders at 20 and 30 days; nonresponders thereafter were excluded.

### 2.3 Data Analysis

FOI responses were collated using Microsoft Excel and descriptive summary statistics produced. Other variables were selected a priori to map against enrolment numbers: number of doctors employed in the National Health Service (NHS), recruitment criteria for internal medicine training (IMT) and postgraduate training competition ratios. These were selected as they each have plausible causal relationships to number of PGCME enrolments and have recorded longitudinal data.

### 2.4 Ethical Approval

Ethical approval was given by the University of Sunderland research ethics committee (ref 032444).

## 3 Findings

### 3.1 Courses Identified

Forty‐one courses were identified meeting the inclusion criteria. Forty of these were at public universities, and one was at a private university.

### 3.2 FOI Responses

FOI requests were sent to all public universities (*n* = 40), as private universities are exempt from FOI legislation. Three universities declined to provide the requested information, citing commercial sensitivity. One university did not respond despite follow‐up communication. The remaining 36 universities (90%) responded with data. All but two universities presented limited data regarding international students, typically reporting ‘< 5’ for each academic year to avoid inadvertent identification of individual students.

The four nonresponding institutions were two in England, one in Wales and one in Northern Ireland; their course titles were conventional variants on *medical* or *clinical education* and did not differ from those at respondent institutions. Their published tuition fees sat toward the lower end of the sector distribution (three of four in the bottom quartile against a sector mean of £3899), which could plausibly bias our enrolment totals in either direction depending on whether lower cost courses attract larger or smaller cohorts than average. The Northern Ireland institution was the only NI‐based PGCME provider we identified, so our enrolment totals contain no Northern Ireland data.

### 3.3 Number of Courses

In 2010–2011, there were at least 13 PGCME courses offered across the UK universities. By 2024–2025, this had risen to 29 courses, representing a 123% increase in availability over the 15‐year period (Table 1).

**TABLE 1:** Number of courses and number of students per year—2010 to 2025.

	Year														
	2010–2011	2011–2012	2012–2013	2013–2014	2014–2015	2015–2016	2016–2017	2017–2018	2018–2019	2019–2020	2020–2021	2021–2022	2022–2023	2023–2024	2024–2025
Number of courses	13	13	15	16	18	17	20	22	25	25	28	28	29	29	29
Number of students	1180	938	1092	1081	1135	960	945	1003	1037	1082	1894	1978	1644	1625	1667

### 3.4 Student Enrolments

**FIGURE 1:**
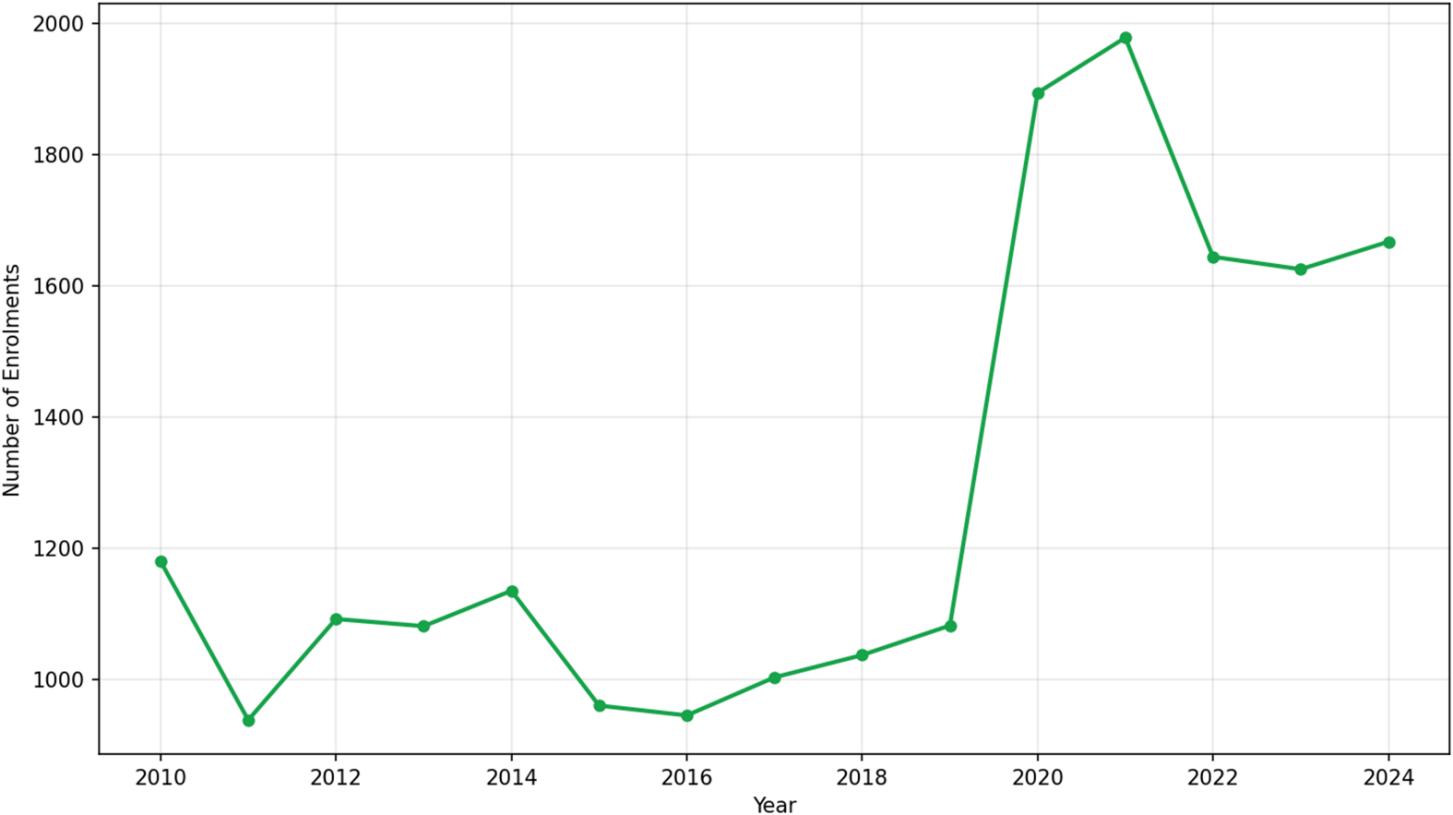
PGCME enrolments per year (2010–2024).

In 2010–2011, the total number of students enrolling on PGCME courses was 1180. In 2024–2025, this figure was 1667, representing a 41% increase. Enrolment numbers remained broadly stable between 2010 and 2019, fluctuating between 938 and 1180 per year. A substantial increase occurred in 2020–2021, when enrolments rose to 1894 (a 75% increase from the previous year). Numbers have since remained elevated, stabilising at approximately 1600–1700 per year (Figure 1).

The mean number of students per course in 2024–2025 was 57.5 (range 4–143).

### 3.5 PGCME Enrolments in Context

To contextualise PGCME enrolment figures, we examined the proportion of the medical workforce pursuing these qualifications and trends in specialty training competition. The UK doctors may apply to specialty training 2 years after graduation from their primary medical degree. Application processes are competitive and typically include scoring of a portfolio of evidence (a structured self‐declared evidence submission scored at application)—academic publications, quality improvement projects and teaching qualifications among other things. IMT and general practice (GP) are the two largest UK specialty training programmes [11] and were used as comparators. This is shown in Table 2.

**TABLE 2:** PGCME enrolments in context: NHS workforce, specialty training competition and application scoring (2010–2024).

Year	NHS England FTE doctors	PGCME enrolments	Enrolments as % of doctors	GP competition ratio	CMT/IMT competition ratio	PGCERT points	Max points	% of max score
2010	97,130	1180	1.21%	1.7	—	—	—	—
2011	98,389	938	0.95%	1.76	—	—	—	—
2012	99,529	1092	1.10%	1.85	—	—	—	—
2013	101,137	1081	1.07%	2.31	2.55	3	64	4.7%
2014	103,330	1135	1.10%	1.62	2.09	3	64	4.7%
2015	104,498	960	0.92%	1.42	1.7	3	64	4.7%
2016	106,131	945	0.89%	1.28	1.53	3	64	4.7%
2017	109,002	1003	0.92%	1.32	1.41	3	64	4.7%
2018	111,247	1037	0.93%	1.33	1.5	3	66	4.5%
2019	116,079	1082	0.93%	1.34	1.43	3	68	4.4%
2020	122,157	1894	1.55%	1.5	1.74	3	68	4.4%
2021	127,319	1978	1.55%	1.79	2.29	3	58	5.2%
2022	131,305	1644	1.25%	2.07	2.45	3	58	5.2%
2023	138,604	1625	1.17%	2.67	2.64	2	40	5.0%
2024	146,387	1667	1.14%	3.67	3.69	3	30	10.0%

Abbreviations: CMT, core medical training; FTE, full‐time equivalent; GP, general practice; IMT, internal medicine training; PGCert, postgraduate certificate.

#### 3.5.1 Data Sources and Geographic Scope

Health policy in the United Kingdom is devolved, and not all data sources used here are pan‐UK. The NHS full‐time equivalent (FTE) doctor numbers [12] describe the English NHS only. Competition ratios (applicants per post) for GP and core medical training (CMT)/IMT were obtained from the NHS England (formerly Health Education England); these describe English recruitment, and Scotland, Wales and Northern Ireland publish separate statistics that are not directly comparable due to differences in workforce definitions and selection processes. GP data for 2010–2012 were derived from archived recruitment figures for the first year of specialty training (ST1); CMT/IMT data before 2013 were unavailable. The IMT scoring framework is also specific to England; historical points awarded for PGCMEs were obtained from archived CMT and IMT recruitment websites via the Internet Archive's Wayback Machine (https://web.archive.org).

We therefore make these comparisons against the English NHS context and treat this as a limitation rather than a pan‐UK analysis. The substantial majority of identified PGCME providers are based in England, so we retained the UK‐wide enrolment totals rather than subsetting to English‐only courses; this decision is revisited in the Limitations section.

#### 3.5.2 Enrolments Relative to the Medical Workforce

Comparing total enrolments against the medical workforce provides a useful benchmark, although PGCME courses also admit other healthcare professionals. In 2010, total enrolments were equivalent to 1.21% of the NHS FTE doctors in England (1180 enrolments; 97,130 doctors). The ratio peaked at 1.55% in 2020–2021 and fell to 1.14% by 2024.

#### 3.5.3 Specialty Training Competition

GP training competition ratios rose from 1.70 applicants per post in 2010 to 3.67 in 2024. CMT/IMT competition ratios increased from 2.55 in 2013 (the first year for which data were available) to 3.69 in 2024.

#### 3.5.4 Value of PGCMEs in Specialty Applications

Between 2013 and 2022, a PGCME awarded 3 points out of a maximum of 58–68 (4.4%–5.2% of the total) for applications to CMT/IMT. In 2024, scoring was restructured: PGCME worth 3 points out of a maximum 30 (10%)—a doubling of the proportional weighting.

#### 3.5.5 Correlations

**FIGURE 2:**
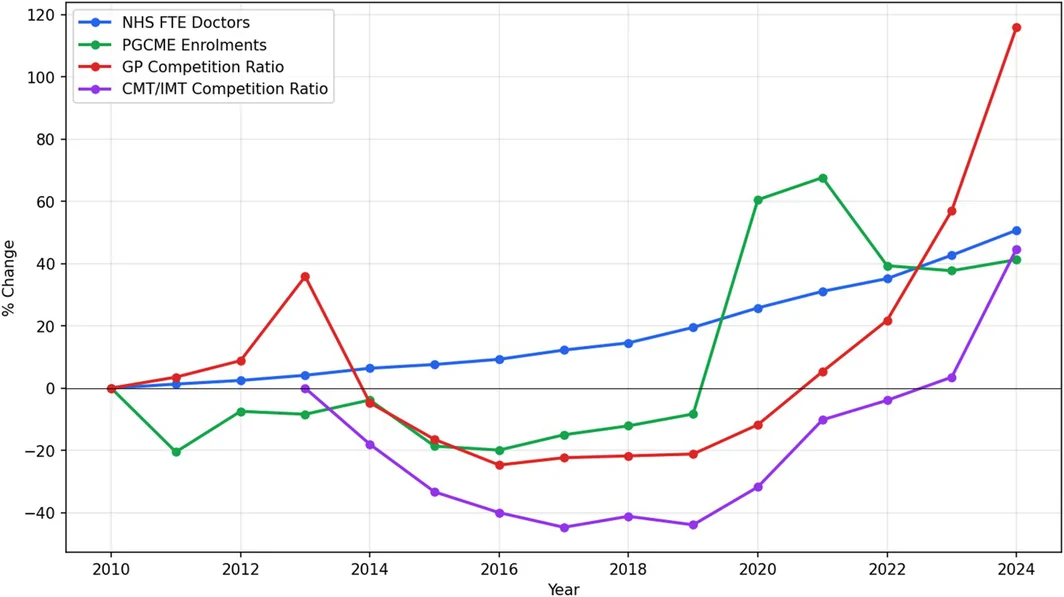
Percentage change in PGCME enrolment, NHS England FTE doctors, GP recruitment competition ratio and CMT/IMT competition ratio since 2010. Abbreviations: CMT, core medical training; FTE, full‐time equivalent; GP, general practice; IMT, internal medicine training; PGCert, postgraduate certificate.

Pearson's correlation coefficients were calculated between PGCME enrolments and three variables: NHS FTE doctors, GP competition ratio and CMT/IMT competition ratio. The NHS FTE doctors showed a strong positive correlation with enrolments (*r* = 0.79, *p* < 0.001); competition ratios showed moderate but nonsignificant correlations (*r* = 0.46–0.53, *p* > 0.05; Table 3 and Figure 2). Enrolments have increased alongside medical workforce growth but are not significantly associated with training programme competitiveness.

**TABLE 3:** Pearson's correlation between PGCME and NHS England full‐time‐equivalent (FTE) doctors, GP competition ratio and CMT/IMT competition ratios.

Variable	*r*	*p*	*R* ^2^
NHS England FTE doctors	0.785	0.0005	0.62
GP competition ratio	0.456	0.088	0.21
CMT/IMT competition ratio	0.532	0.075	0.28

Abbreviations: CMT, core medical training; GP, general practice; IMT, internal medicine training.

## 4 Discussion

### 4.1 Summary of Findings

No published data on PGCME enrolment existed before this study. We found that courses more than doubled between 2010 and 2024 (13–29), while student numbers only rose by 41% (1180–1667). Enrolments stabilised through the 2010s, spiked in 2020–2021, then plateaued. Cohort sizes ranged from 4 to 143.

These numbers describe a sector that has grown substantially. But growth in enrolments does not, by itself, tell us whether PGCMEs are professionalising the medical education workforce or simply becoming another portfolio requirement or whether other forces are at work.

### 4.2 The 2020 Spike

Enrolments jumped sharply in 2020–2021. Several factors likely converged.

COVID‐19 moved many courses online [13], making them accessible to clinicians who could not previously travel to campus, and pushed educators to formalise skills they were acquiring on the job. The spike also coincided with the first full year after the 2019 IMT scoring reforms made teaching qualifications a distinct category, against rising competition (IMT ratios climbed from 1.43 in 2019 to 1.74 in 2020). Stabilisation since 2021–2022 suggests a new baseline rather than a return to prepandemic levels.

### 4.3 Professionalisation or Credentialism?

Two narratives might explain PGCME growth. In the professionalisation narrative, doctors enrol to become better educators. In the credentialism narrative, doctors enrol because lacking the qualification limits competitive options, not because they plan sustained educational work.

A third possibility is that universities have expanded provision for financial reasons independent of workforce need: Postgraduate certificates generate fee income with modest delivery costs, particularly when taught asynchronously.

These narratives are not mutually exclusive. Some students enrol for development and benefit from the credential; others enrol for the credential and discover a vocation. But the balance matters: If credentialism dominates, enrolment growth may not translate into a more skilled educational workforce.

Several features of the data lean toward credentialism.

### 4.4 Credential Inflation

PGCMEs matter more for specialty training applications than ever before. The UK training programmes award portfolio points for teaching qualifications, and the scoring rules have shifted in ways that favour PGCMEs.

In IMT, teaching qualifications were originally counted under ‘postgraduate qualifications’ alongside research degrees. From 2019, ‘training in teaching’ became a separate category, making PGCMEs a primary route to teaching points rather than one option among several. In 2024, the total points available fell from 40 to 30, while PGCME points rose from 2 to 3, meaning teaching qualifications now represent 10% of the portfolio score, up from 4.7% a decade earlier.

This increased value may be driving credential inflation. As competition intensifies, many applicants acquire PGCMEs, but as the qualification becomes more common, lacking one may penalise applicants more than holding one distinguishes them.

The 2024 scoring change compounds this: PGCert, PGDip, and Masters now score identically (3 points), removing any incentive to progress beyond certificate level. Other specialties retain graduated scoring (e.g., Anaesthetics: 3/5/6 for PGCert/PGDip/Masters), but IMT's flattened scoring affects a substantial proportion of applicants.

### 4.5 Enrolments Track Workforce Growth

Against the credentialism narrative sits one reassuring pattern. PGCME numbers grew 41% over the study period (1180 to 1667), while the NHS medical workforce grew 51% (97,130 to 146,387 FTE). Enrolments per doctor therefore fell slightly, from 1.21% in 2010 to 1.14% in 2024, having stayed within a 0.9%–1.6% band throughout. If this broad stability holds, providers planning capacity can use workforce projections under the NHS Long Term Workforce Plan [14] as a proxy for demand.

But proportional growth does not resolve the underlying question. The absolute number of PGCME holders has increased substantially, and if PGCMEs are simply being used to tick a box, their effectiveness at developing the workforce is questionable. Aggregate data cannot tell us.

### 4.6 The Teaching Fellow Question

If credentialism is a driver, who are the credentialists? Clinical teaching fellows (CTFs) are one plausible group.

The CTF role has expanded in recent years [15]. CTFs are doctors who step off the training pathway, typically for a year before specialty applications, to combine undergraduate teaching with some clinical work. Many NHS Trusts (local NHS provider organisations) fund their CTFs to complete a PGCME as part of the post.

CTF expansion tracks medical student expansion. This creates a predictable annual cohort of PGCME students, largely trust‐funded and largely transient. Most CTFs return to specialty training after a year. CTFs themselves report teaching interest as their dominant motivation, with CV‐building a minority concern [16]; their supervisors are more direct, describing the qualification as substantively weighting subsequent specialty applications [17].

### 4.7 A Fragmented Market

Twenty‐nine courses now compete for roughly 1600 students per year. Some enrol fewer than 10; others exceed 140. Fees range from under £2500 to over £5000, and delivery modes vary from fully asynchronous to in‐person; whether students select on cost, convenience or brand is unknown. This variation raises questions the data cannot answer.

Business models also vary: Large asynchronous courses can generate substantial fee income with limited staffing costs. Whether institutions offering this model pursue educational mission or passive revenue is not apparent from enrolment figures, but the question matters for course quality.

### 4.8 Beyond Medicine, Beyond the United Kingdom

Although our data are UK‐specific, the dynamics they describe are not. The mechanism we propose—that competitive selection scoring of teaching qualifications may create demand independent of intent to teach—should be visible in any system that ties teaching credentials to selection. Analogous patterns have been documented in the Australian specialty trainees acquiring postgraduate qualifications beyond clinical need [7], and the North American Health Professions Education programmes have grown in number substantially over the last 30 years [18]. Equivalent pressures apply to nursing and allied‐health educators in many jurisdictions. We therefore offer the UK case not as the primary phenomenon but as a tractable system for measuring credentialing scale, and encourage analogous studies in other systems and professions.

## 5 Limitations

Our contextual comparators describe the English NHS only, so enrolment‐to‐workforce and competition‐ratio comparisons should be read as English‐context indicators rather than pan‐UK estimates. The absence of the Northern Ireland enrolment data (see FOI responses) compounds this.

FOI requests yield aggregate counts. We know how many students enrolled; we do not know who they were, why they enrolled, how they funded their studies, whether they completed or what they did afterwards. Because the data do not break down students by profession, our comparison to the NHS medical workforce assumes all enrolees are doctors, meaning these percentages represent an upper estimate. This limits direct testing of the credentialism hypothesis.

Most universities suppressed international student data, leaving the international dimension unassessable. Finally, the hypotheses we advance (COVID accessibility, scoring reforms, credential inflation, CTF growth) remain untested.

## 6 Where Next?

This study establishes baseline data on a sector that has grown without systematic monitoring. But baseline data cannot answer the question that motivated the Introduction: Is this growth professionalising the medical education workforce?

Future research should address who enrols (CTFs, consultants, other professions; self‐funded versus employer‐funded), how students choose courses (price, convenience, prestige, quality), whether the qualification changes practice (sustained educational engagement, teaching skill, leadership progression) and how new courses fare against established competitors. If most holders never teach substantially after their fellowship year, the sector may be producing credentials rather than educators.

One concrete next step is the UK Medical Education Database (UKMED), which links Higher Education Statistics Agency (HESA) records to General Medical Council training data, online training applications and National Training Survey responses [19]. Extending UKMED's HESA extract to capture postgraduate Level 7 (Master's‐level) enrolment in medical or clinical education would enable individual‐level analysis of who enrols, completion rates and downstream outcomes including specialty success and longer‐term educational role.

Policy implications follow. If PGCME provision is to support the NHS Long Term Workforce Plan, policymakers need to know whether the current fragmented market matches geographic and specialty need and whether coordination, regional incentives or quality standards beyond AoME accreditation are warranted.

## 7 Conclusion

This study provides the first national data on PGCME enrolment in the United Kingdom. Over 15 years, courses more than doubled, while student numbers grew more slowly than the medical workforce. The 2020–2021 spike likely reflects pandemic‐related accessibility and scoring reforms rather than lasting demand growth. Whether this growth is producing skilled educators, portfolio‐ready applicants or both remains unknown—and answering that is important for those planning educator training and for the clinical teachers investing in these qualifications.

*Over 15 years, courses more than doubled while, student numbers grew more slowly than the medical workforce*.

## Author Contributions

**Matthew Bowker:** conceptualization, investigation, writing – original draft, methodology, data curation. **Leah Williams:** investigation, conceptualization, methodology, writing – original draft. **Michael Atkinson:** writing – review and editing, conceptualization. **Chris Tiplady:** writing – review and editing, conceptualization.

## Funding

The authors have nothing to report.

## Conflicts of Interest

All authors are faculty for the postgraduate Medical Education courses at the University of Sunderland.

## Data Availability

The data that support the findings of this study are available from the corresponding author upon reasonable request.
